# Predictors of Death during Tuberculosis Treatment in TB/HIV Co-Infected Patients in Malaysia

**DOI:** 10.1371/journal.pone.0073250

**Published:** 2013-08-12

**Authors:** Ismawati Ismail, Awang Bulgiba

**Affiliations:** 1 Julius Centre University of Malaya, Department of Social and Preventive Medicine, Faculty of Medicine, University of Malaya, Kuala Lumpur, Malaysia; 2 Ministry of Health, Putrajaya, Malaysia; Institute of Infectious Diseases and Molecular Medicine, South Africa

## Abstract

**Background:**

Mortality among TB/HIV co-infected patients is still high particularly in developing countries. This study aimed to determine the predictors of death in TB/HIV co-infected patients during TB treatment.

**Methods:**

We reviewed medical records at the time of TB diagnosis and subsequent follow-up of all newly registered TB patients with HIV co-infection at TB clinics in the Institute of Respiratory Medicine and three public hospitals in the Klang Valley between January 2010 and September 2010. We reviewed these medical records again twelve months after their initial diagnosis to determine treatment outcomes and survival. We analysed using Kaplan-Meier and conducted multivariate Cox proportional hazards analysis to identify predictors of death during TB treatment in TB/HIV co-infected patients.

**Results:**

Of the 227 patients studied, 53 (23.3%) had died at the end of the study with 40% of deaths within two months of TB diagnosis. Survival at 2, 6 and 12 months after initiating TB treatment were 90.7%, 82.8% and 78.8% respectively. After adjusting for other factors, death in TB/HIV co-infected patients was associated with being Malay (aHR 4.48; 95%CI 1.73-11.64), CD4 T-lymphocytes count < 200 cells/µl (aHR 3.89; 95% CI 1.20-12.63), three or more opportunistic infections (aHR 3.61; 95% CI 1.04-12.55), not receiving antiretroviral therapy (aHR 3.21; 95% CI 1.76-5.85) and increase per 10^3^ total white blood cell count per microliter (aHR 1.12; 95% CI 1.05-1.20)

**Conclusion:**

TB/HIV co-infected patients had a high case fatality rate during TB treatment. Initiation of antiretroviral therapy in these patients can improve survival by restoring immune function and preventing opportunistic infections.

## Introduction

Tuberculosis and human immunodeficiency virus (TB/HIV) co-infection is an important global public health problem. TB is the most common opportunistic infection and the leading cause of death in HIV-infected patients [1]. Globally, there were an estimated 8.7 million incident cases of TB in 2011 with 13% were co-infected with HIV. There were almost a million deaths among HIV-negative TB patients with an additional 0.43 million deaths reported among HIV-positive TB patients [2].

Malaysia is categorized by the World Health Organization (WHO) as an intermediate TB burden country [3]. As in other developed and industrialized countries, the TB problem in Malaysia had declined significantly between 1970 and 1980. Factors that have contributed to the reduction in TB incidence include improvements in socioeconomic status, better ventilation of homes and work sites; and an improved health system. However, from early 1995, the incidence of tuberculosis has slowly increased from an incidence rate of 58 per 100,000 in 1995 to 72 per 100,000 in 2011. Several factors were responsible for the increasing TB incidence including the HIV infection, influx of immigrants from endemic neighbouring countries, increased in urban migration and drug abuse. The HIV epidemic reinforces the need to focus on the identification and cure of infectious TB patients [4].

The present study reports on the survival of a cohort of HIV-positive tuberculosis patients registered in 2010 in the central region of Malaysia. In Malaysia, there is still a gap in knowledge in the understanding of TB/HIV co-infection particularly on the survival and predictors of death in TB-HIV co-infected patients. Most of the studies on TB/HIV survival were conducted in African countries. Some studies have been published recently in neighbouring countries such as Thailand and Vietnam [5–8]. There are only a few articles regarding characteristics of TB/HIV co-infected patients in selected states in Malaysia [9,10]; but none has reported on the survival and predictors of death. The aim of this study was to evaluate the survival time and predictors of death in Malaysian HIV-infected tuberculosis patients.

## Study Population and Methods

Patients who were registered for TB treatment between January 2010 and September 2010; and who were documented to have a concomitant HIV infection in the Institute of Respiratory Medicine and three public hospitals in Selangor (Kajang Hospital, Klang Hospital and Sungai Buloh Hospital) between January 2010 and September 2010 were included in the study.

In Malaysia, all TB patients are routinely screened for HIV, based on the guidelines of the Ministry of Health in 2002 [11]. The HIV test was done at the time of TB diagnosis. HIV enzyme-linked immunosorbent assay (ELISA) test results for these patients were traced and those who had positive results included in the study.

Inpatient and outpatient medical records as well as TB Information System (TBIS) documents were reviewed to obtain all the dependent and independent variables including information on socio-demographic data, lifestyle factors, clinical characteristics and laboratory profiles. The information gathered was used to complete a standard data collection form. To ensure confidentiality, each case was assigned a study identification number that was included on all records as personal identifiers.

Patients who were transferred out to other treatment centres or had their initial TB diagnosis changed to other diagnosis by the attending physician were excluded from the study. Then the patients’ survival status and their survival times were determined. For this purpose, patients were followed up until 30^th^ September 2011.

There were also patients who had either defaulted treatment or were lost to follow-up. In these patients, their mortality or survival status was obtained by contacting the patients or relatives through the phone number or address as recorded in their medical records. We confirmed their survival status through the National Registration Department. We further checked their names against the Electoral Register of 2011 to confirm their mortality status.

We defined the survival time as the time from TB diagnosis to death, which is the interval between the date on which TB was diagnosed and the date of death. Patients were censored at the date of the last visit if they were lost to follow-up. All patients who survived were censored from the study on 30 September 2011. We classified any death that occurred during TB treatment as TB death, consistent with WHO guidelines [12]. We also analysed the predictors of death in TB/HIV co-infected patients. Sociodemographic characteristics that were investigated included age, gender, ethnicity, nationality, employment status, marital status and incarceration. Lifestyle characteristics were: smoking status, alcohol intake, mode of HIV transmission and body weight. Clinical characteristics were: types of TB, status of TB diagnosis, TB symptoms at presentation (fever, cough with sputum, night sweat), changed in TB treatment, co-morbidity with diabetes mellitus, number of opportunistic infections and status of antiretroviral therapy. Laboratory characteristics were: baseline haemoglobin level, total white blood cell, serum albumin, CD4 counts, HbsAg, toxoplasma serology and anti-HCV serology.

### Statistical analysis

We entered the data and analysed it using SPSS 17.0. All continuous variables were described using means and standard deviations while categorical variables were described using counts and proportions (%). The Kaplan-Meier technique was employed to plot the survival graph. We used Cox proportional hazard regression analysis to identify independent predictors of death. We checked for interaction and adjusted for confounding. We selected the final model based on the principle of parsimony and best fit using the Hosmer-Lemeshow approach of using -2 log likelihood ratios. We presented the results in the form of hazard ratios (HR) and 95% confidence intervals (CI). The level of significance was set at 0.05.

### Ethics

Ethical clearance was obtained from the Medical Ethics Committee of University of Malaya (MEC Ref No. 776.11) and from the Medical Review and Ethics Committee (MREC), Ministry of Health Malaysia with the reference number NMRR-10-1201-6599. The requirement to obtain informed written consent from each individual was waived by the Medical Review and Ethics Committee, Ministry of Health Malaysia as the study was limited to review of existing medical records.

## Results

### Socio-demographic characteristics

A total of 2262 tuberculosis patients were registered between 1^st^ January 2010 and 30^th^ September 2010 in Hospital Sungai Buloh, Hospital Kajang, Hospital Tuanku Ampuan Rahimah, Klang; and Institute of Respiratory Medicine, Kuala Lumpur. Among them, 267 patients (11.8%) were co-infected with HIV. Of these 267, 32 (12.0%) had been transferred out to other treatment centres and 8 (3.0%) had their diagnoses changed because they were later found to have a different diagnosis. This meant that a total of 40 patients had to be excluded from the study, leaving 227 patients eligible for analysis.

The mean age of TB/HIV co-infected patients were 39.1 (SD 8.6) years old and ranged from 16 to 62 years. The peak age group was 35 to 54 years old (62.1%). Male to female ratio was 7:1. Ethnic Malays constituted the highest proportion (48.5%) of patients followed by Chinese (16.3%), Indians (16.3%) and Others (18.9%). The majority of them were Malaysians (n=185, 81.5%) while non-Malaysians who originated from Myanmar, Indonesia, Nepal and Thailand constituted 18.5% (n=42). More than half the patients were unemployed (57.3%, n=130). The majority of TB/HIV co-infected patients were single or divorced (67.0%, n=152).

### Survival curve and predictors of death

To determine the survival of TB/HIV co-infected patients during TB treatment, the survival status of all patients (n = 227), including those who were still on TB treatment were assessed at the end of the study period. Patients whose treatment were interrupted for two months or more were classified as ‘treatment defaulted’. At the end of the study period, 25.6% (n=56) had defaulted treatment. To verify the patient’s status, patients data were linked to the National Registration Department database. Patients who died during treatment default were reclassified as having died instead of having defaulted treatment. In total, seven (7) patients who were originally classified as defaulters were later reclassified as having died, giving the total number of deaths during TB treatment to be 53 (23.3%).

**Figure 1:**
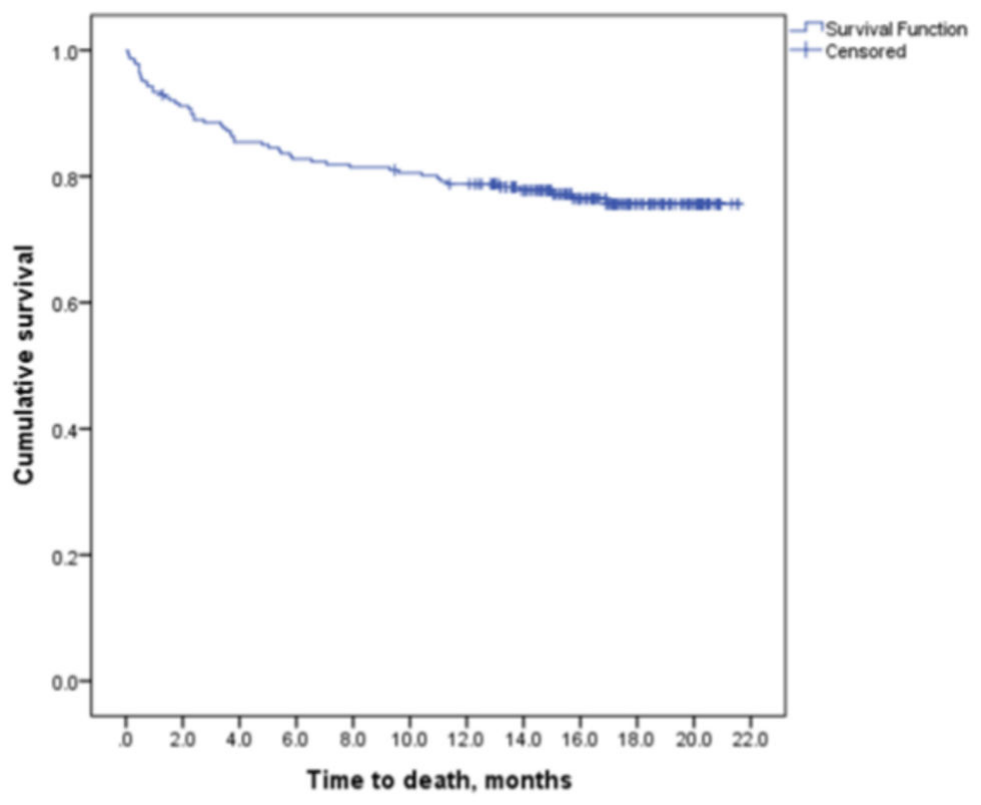
Kaplan-Meier survival curve of TB/HIV co-infected patients (n=227).

The mean follow-up duration was 14.3 months (SD 6.3) months and the median was 15.9 (IQR 5.8) months. Survival at 2, 6 and 12 months after initiating TB treatment were 90.7% (95% CI: 90.3-91.1), 82.8% (95% CI: 82.6-83.1) and 78.8% (95% CI: 78.3-79.3) respectively. The survival curve becomes much flatter after 6-months of TB treatment (Figure **1**).

Table 1 displays the frequency and univariate Cox regression analyses of sociodemographic and lifestyle characteristics in TB/HIV co-infected patients. Table 2 displays the frequency and univariate Cox regression analyses of clinical and laboratory characteristics in TB/HIV co-infected patients. The log rank p value less than 0.25 was set as cut off value to choose predictors for entry into multiple Cox proportional hazards regression analysis. The cut off of p less than 0.25 was chosen in order not to miss significant predictors which may have been confounded during univariate analysis. The outcome is a single outcome variable with two categories (alive or dead). There were seventeen predictors with a log rank p-value of less than 0.25 - age group, marital status, ethnicity, employment status, incarceration, body weight, cough with sputum at diagnosis, night sweats, co-morbidity with diabetes mellitus, number of opportunistic infections, antiretroviral therapy (ART), haemoglobin level, serum albumin level, total white blood cell count, toxoplasma serology test, anti-HCV serology and CD4 T-lymphocytes counts. During the multivariate analysis analysis using Cox proportional hazards regression model; ethnicity, number of opportunistic infections, anti-retroviral therapy, total white blood cell count (WBC) and CD4 T-lymphocytes were found to be associated with death in TB/HIV co-infected patients (Table 3).

**Table 1:** Frequency and univariate Cox regression analyses of socio-demographic and lifestyle characteristics in 227 TB/HIV co-infected patients.

**Variables**	**N (%)**		**HR (95%CI)**		***p*-value**
**Age group, year**					
≤ 34		73 (32.2)	1		
35-54		141 (62.1)	1.70 (0.89, 3.26)		0.108
≥ 55		13 (5.7)	1.48 (0.42, 5.24)		0.545
**Age (mean (SD))**		39.1 + 8.6 years			
**Gender**					
Male		200 (88.1)	1.01 (0.43, 2.35)		0.992
Female		27 (11.9)	1		
**Ethnicity**					
Malay		110 (48.5)	2.90 (1.14,7.41)		0.026
Chinese		37 (16.3)	1.17 (0.34,4.03)		0.807
Indian		37 (16.3)	1.89 (0.62,5.77)		0.266
Others		43 (18.9)	1		
**Nationality**					
Malaysian		185 (81.5)	1.75 (0.40, 7.76)		0.280
Non- Malaysian		42 (18.5)	1		
**Employment status**					
Employed		97 (42.7)	1		
Unemployed		130 (57.3)	0.39 (0.21, 0.73)		0.003
**Marital status**					
Single/divorced		152 (67.0)	1.78 (0.94, 3.39)		0.079
Married		75 (33.0)	1		
**Incarceration** ^a^					
Yes		21 (18.5)	0.39 (0.20, 0.78)		0.008
No		206 (81.5)	1		
**Smoking status**					
Smoker		168 (74.0)	0.72 (0.37,1.39)		0.325
Non-smoker		59 (26.0)	1		
**Alcohol intake**					
No		173 (76.2)	0.91 (0.46,1.86)		0.777
Yes		54 (23.8)	1		
**Mode of HIV transmission**					
IV^b^ drug users		127 (55.9)	1.08 (0.33, 3.48)		0.902
Sexual		89 (39.6)	0.35 (0.10,1.27)		0.311
Others^c^		11 (4.5)	1		
**Body weight (Mean (SD))**		49.7 (±9.0)kg	0.97 (0.94-1.00)		0.059

^a^Incarceration: Prisoners/Drug Rehabilitation Centre inmates at the time of TB diagnosis.

^b^IV:intravenous

^c^Others: Blood transfusion (n=1) and Unknown (n=10)

**Table 2:** Frequency and univariate Cox regression analyses of clinical and laboratory characteristics in 227 TB/HIV co-infected patients.

**Variables**	**N (%)**		**HR (95%CI)**	***p*-value**
**Types of TB**				
Smear positive PTB	92 (40.5)		1.02 (0.53, 1.97)	0.944
Smear negative PTB	71 (31.3)		0.94 (0.46, 1.90)	0.857
Extrapulmonary TB	64 (28.2)		1	
**Status of TB diagnosis**				
Newly diagnosed TB	161 (70.9)		0.85 (0.48,1.51)	0.575
Ever had TB before		66 (29.1)	1	
**Fever**				
Yes	170 (74.9)		1.04 (0.56-1.94)	0.904
No	57 (25.1)		1	
**Cough with sputum**				
Yes	110 (48.5)		1.63 (0.94, 2.81)	0.081
No	117 (51.5)		1	
**Night sweats**				
Yes	98 (43.2)		0.64 (0.36, 1.13)	0.125
No	129 (56.8)		1	
**Changed in TB treatment^a^**				
Yes	37 (16.4)		0.99 (0.48, 2.03)	0.982
No	190 (83.7)		1	
**Diabetes Mellitus**				
Yes	6 (2.6)		0.44 (0.14, 1.40)	0.165
No	221 (97.4)		1	
**Number of OI^b^**				
No OI	119 (52.4)		1	
One OI	79 (34.8)		1.86 (1.02, 3.39)	0.430
Two OIs	23 (10.1)		2.02 (0.85,4.78)	0.110
Three or more OIs	6 (2.6)		3.72 (1.10,12.52)	0.340
**Anti-retroviral therapy**				
Yes	140 (61.7)		1	
No	87 (38.3)		2.50 (1.45, 4.31)	0.001
**Hemoglobin level**				
< 12 g/dL	178 (78.4)		1.60 (0.75,3.40)	0.221
≥ 12 g/dL	49 (21.6)		1	
**Total White Blood Cells**				
**(Mean (SD))**	6.3 (± 3.3) 10^3^/µl		1.12 (1.05-1.18)	< 0.001
**Serum albumin**				
**(Mean (SD))**	26.5 (± 7.5) g/L		0.90 (0.86, 0.94)	< 0.001
**CD4 count**				
Not available	168 (74.0)		3.95 (0.94, 16.53)	0.060
< 200 cells/µl	39 (17.2)		3.90 (1.21,12.54)	0.023
≥ 200 cells/µl			1	
**HbsAg**				
Reactive	15 (6.6)		0.82 (0.26-2.63)	0.737
Non-reactive/unknown	212 (93.4)		1	
** *Toxoplasma* serology test**				
Reactive	51 (22.5)		1.77 (0.99, 3.15)	0.053
Non-reactive/unknown	176 (77.5)		1	
**Anti-HCV serology**				
Reactive	127 (55.9)		1.73 (0.97, 3.08)	0.062
Non-reactive/unknown	89 (39.2)		1	

^a^Changed in treatment: change from the initial TB regime (HRZ) to another due to side effects of treatment or adverse reaction.

^b^OI: Opportunistic infection

**Table 3:** Final Cox regression model predictors of death in TB/HIV co-infected patients.

**Characteristics**	**Adjusted HR^a^ **	**95%CI**	***p*-value**
**Ethnicity**			
Malay	4.48	1.73-11.64	0.002
Chinese	1.78	0.51-6.25	0.370
Indian	2.40	0.76-7.56	0.134
Others	1.00	-	
**CD4 T-lymphocytes count**			
Not available	2.21	0.50-9.81	0.299
< 200 cells/µl	3.89	1.20-12.63	0.024
≥ 200 cells/µl	1.00	-	
**Number of Opportunistic infections (OI)**			
No OI	1.00	-	
One OI	2.68	1.40-5.13	0.003
Two OIs	3.32	1.33-8.29	0.010
Three or more OIs	3.61	1.04-12.55	0.044
**Anti-retroviral therapy**			
Yes	1.00	-	
No	3.21	1.76-5.85*	<0.001
**Total White Blood Cells**	1.12	1.05-1.20*	0.001

HR: Hazards Ratio, CI: Confidence Interval. ^a^Adjusted for age group, marital status, employment status, incarceration, body weight, cough with sputum at diagnosis, night sweats, co-morbidity with diabetes mellitus, haemoglobin level, serum albumin level, toxoplasma serology test and anti-HCV serology.

Ethnicity is the strongest risk factor for death in this study. The risk of dying in Malays is almost five times higher than ethnic group classified as ‘Others’ (HR: 4.48; 95%CI: 1.73-11.64).

Low CD4 T-lymphocytes count is the strongest clinical predictor of death in TB/HIV co-infected patients. Patients with CD4 T-lymphocytes count less than 200 cells/µl had almost four times higher risk of death compared to CD4 T-lymphocytes count more than 200 cells/µl (HR 3.89; 95% CI: 1.20-12.63). Patients with three or more opportunistic infections had 3.61 times higher risk of death than patients who do not have any opportunistic infection (HR: 3.61; 95%CI: 1.04-12.55). The risk of death in patients who were not on antiretroviral therapy was 3.21 times compared to patients on antiretroviral therapy (HR: 3.21; 95%CI: 1.76-5.85). For every 10^3^ cells per microliter unit increase in total white blood cell, the risk of death increased by 12% (HR: 1.12; 95%CI: 1.05-1.20).

## Discussion

TB is a common opportunistic infection and cause of death in HIV-infected patients in many parts of the world, particularly in developing countries. Results in our study have estimated the prevalence of TB/HIV in Klang Valley is 11.0% which is slightly higher than the national prevalence of HIV-infected patients among tested TB patients in Malaysia (7.2%) in 2010 [2]. HIV prevalence in TB patients in Asia ranged from 0.9% to 38% in 2010 [2]. TB/HIV co-infection is still relatively low in Malaysia compared to other countries in Asia.

Data from the National TB Control Programme showed that the rate of TB death in Malaysia in 2011 was 8.0% of the total notified TB cases in that year [13]. Our study denotes that HIV increases the risk of death in our patient cohort. HIV infection as a predictor of death in TB patients has been established in many previous studies [14–19]. In this study, death during TB treatment occurred in 53 (23.3%) of TB/HIV co-infected patients with 40% of deaths occurring within two months of TB diagnosis. This finding is similar to case fatality rates during TB treatment that was reported in previous studies in Thailand, which was 29% [5] and Vietnam (26%) [8].

Ethnicity is the only social risk factor for survival in this study. Malays had almost five times higher risk of death than the ethnic group classified as ‘Others’ (HR: 4.48; 95%CI: 1.73-11.64) even after adjusting for potential confounders including employment status, body weight and serum albumin level in multivariate analysis. This warrants further research as reasons for this are not very clear. Future research might investigate the ethnic differences in relation to treatment adherence and access to care.

The finding that every 10^3^ increase in total white blood cell per microliter (HR: 1.12; 95%CI: 1.05-1.20) is associated with higher mortality in HIV-infected TB patients finding is novel. We have not found any studies in the literature that present a similar association. Most previous studies have focused on T lymphocyte subsets, particularly CD4^+^, and generally reported depressed CD4^+^ T cells in peripheral blood of TB patient. In this study, the total white blood cell results were that documented at the time of TB diagnosis (pre-TB treatment). This finding may reflect that the risk of dying is increased with the severity of infection at TB diagnosis.

The immune responses against HIV-infected tuberculosis have not been fully clarified. Generally, infection will cause white blood cells to be elevated. The major types of white blood cells; neutrophils, lymphocytes, monocytes, eosinophils and basophils play a different role in the immune system with a different disease-fighting activity. In active TB disease, neutrophils and monocytes are the main components of white blood cell that response to mycobacterial infection [20]. A recent study by Berry et al supported that neutrophils play a role in the pathogenesis of TB which resulted from over-activation by IFN-gamma and type I IFNs [21]. A study in South Africa found that patients at TB diagnosis had a high absolute neutrophil and monocyte counts which depleted during treatment but low lymphocyte subset counts which increased with TB treatment [22]. However, in patients who were co-infected with HIV, HIV viruses will attack lymphocytes and causing further depletion of CD4^+^ T cells. There is conflicting evidence on the effect of *Mycobacterium tuberculosis* (MTB) on HIV replication but several studies shown that MTB infection may increase HIV replication and worsen the patient’s immune status [23].

Another factor that is independently associated with decreased survival in this study is the number of opportunistic infections. Patients with three or more types of opportunistic infections have 3.61 times higher risk of death than patients who do not have any opportunistic infection (HR: 3.61; 95% CI: 1.04-12.55). The finding that opportunistic infection is associated with death were consistent with other studies in this area [24–28]. Although these studies demonstrated a positive association between opportunistic infections and death in TB/HIV co-infected patients, they did not examine the relationship between the number of opportunistic infections and risk of death. To our knowledge, this is the first study to report that the number of opportunistic infections is associated with death in TB/HIV co-infection. TB is known to be the most common opportunistic infections in HIV patients. A study by Wong et al in South Africa who investigated the cause of death on antiretroviral therapy by needle biopsy found that TB was the main cause of death regardless of antiretroviral status (pre-ART, early-ART or late-ART); with disseminated TB responsible for 87% of deaths in the first three months of ART [29].

In addition to concurrent opportunistic infections, not receiving antiretrovirals (HR: 3.21; 95% CI: 1.76-5.85) is also associated with death in TB/HIV co-infected patients. This study demonstrated that ART can improve both the TB treatment outcome and also survival. The mean survival time of patients on ART is 19.0 months (95% CI: 18.1-20.0) and the mean survival time of patients who were not on ART is 15.1 months (95% CI: 13.2-17.0). The survival probability at 12 months for patients on ART is 86.3% compared to 66.7% in patients who were not on ART. This finding shows that HIV-infected TB patients treated with ART had almost 23% better survival compared with those who were not treated with ART. The benefit of ART in reducing the death rates of TB/HIV co-infected patients have been well documented in other studies [5,7,30–32]. The widespread use of antiretroviral therapy since 1996 has markedly improved the survival of HIV-infected patients both in developing and developed countries by reducing the number of deaths from many opportunistic infections. In TB/HIV co-infected patients, to introduce antiretroviral therapy is not an easy decision because of concerns about immune reconstitution inflammatory syndrome (IRIS). However, Velasco et al proved that HIV-associated TB patients will have better survival if HAART and TB treatment were started concurrently (aHR 0.37, 95% CI 0.17–0.66) [33].

There have been clinical trials that show timely initiation of antiretroviral therapy in TB/HIV co-infected patients save lives particularly those with low CD4 [34–37]. A recent clinical trial by Abdool Karim et al., comparing the outcomes in early versus late ART concluded that in severely immune-compromised patients with CD4 counts less than 50/mm^3^, ART should be started as soon as possible after the start of TB treatment [38]. Although earlier ART is associated with a higher risk of IRIS, but the finding that it is also associated with two thirds lower risk of death than later ART is far more important (incidence rate ratio, 4.7). However, for those with CD4 counts higher than 50/mm^3^, they recommended that the initiation of ART be deferred until the first four weeks of continuation phase of TB treatment in order to reduce the risk of IRIS [38].

This study adds to the literature that low CD4 T-lymphocytes are associated with higher risk of death in TB/HIV co-infected patients during TB treatment. In our study, patients with CD4 T-lymphocytes count lower than 200 cells/µl had almost four times higher risk of death compared to those with CD4 T-lymphocytes count more than 200 cells/µl (HR 3.89; 95% CI: 1.20-12.63). This finding is well established in other previous studies [5,24,25,30,31,33,39,40]. The mean survival time of patients with CD4 < 200 cells/µl is 17.0 months (95% CI: 15.8-18.2) which is lower than the mean survival time for patients with CD4 > 200 cells/µl (20.0 months; 95% CI: 18.7-21.1). The mean survival time for patients whose CD4 counts are not available is 16.1 months (95% CI: 12.5-19.8) which is similar to those with CD4 < 200 cells/µl; suggesting that this group of patients might have had lower CD4 counts as well. Even though opportunistic infections usually occur at less than 200CD4+ cells/µl, pulmonary TB is commonly thought to occur at a higher concentration [41].

This study is limited by its observational study design. In this study, some of the predictors that had been related to observed survival in other studies of TB/HIV co-infected patients such as Directly Observed Treatment (DOT) supervision, Tuberculin skin testing (TST), co-trimoxazole preventive therapy; and complications regarding treatment were not assessed. It would also interesting to know the differential for the WBC particularly the absolute neutrophil count. However, these predictors were excluded from the analysis because data related to these predictors were incomplete or unavailable in many patients’ record.

This is the first study to explore the risk of death during TB treatment in TB/HIV co-infected patients in Malaysia. A better understanding of the predictors of death among Malaysians could provide opportunities for policy makers to estimate the burden of HIV infection among active TB cases in Malaysia; thus enabling informed decisions on priorities. The results of this study will also help to engage policy makers, researchers and communities in working together to generate the knowledge that is needed for better care for TB/HIV co-infected patients in Malaysia; as well as to promote further research in this area.
